# Pore-scale observations of natural hydrate-bearing sediments via pressure core sub-coring and micro-CT scanning

**DOI:** 10.1038/s41598-022-07184-6

**Published:** 2022-03-02

**Authors:** Liang Lei, Taehyung Park, Karl Jarvis, Lingli Pan, Imgenur Tepecik, Yumeng Zhao, Zhuan Ge, Jeong-Hoon Choi, Xuerui Gai, Sergio Andres Galindo-Torres, Ray Boswell, Sheng Dai, Yongkoo Seol

**Affiliations:** 1 National Energy Technology Laboratory, U.S. Department of Energy, Morgantown, WV 26507 USA; 2 Key Laboratory of Coastal Environment and Resources of Zhejiang Province (KLaCER), School of Engineering, Westlake University, Hangzhou, 310024 Zhejiang Province China; 3 Institute of Advanced Technology, Westlake Institute for Advanced Study, Hangzhou, 310024 Zhejiang Province China; 4 Geosystems Engineering, Georgia Institute of Technology, Atlanta, GA 30332 USA; 5 Leidos Research Support Team, Morgantown, WV 26507 USA; 6 National Energy Technology Laboratory, U.S. Department of Energy, Pittsburgh, PA 15236 USA

**Keywords:** Solid Earth sciences, Geophysics, Petrology, Sedimentology

## Abstract

Both intra-pore hydrate morphology and inter-pore hydrate distribution influence the physical properties of hydrate-bearing sediments, yet there has been no pore-scale observations of hydrate habit under pressure in preserved pressure core samples so far. We present for the first time a pore-scale micro-CT study of natural hydrate-bearing cores that were acquired from Green Canyon Block 955 in UT-GOM2-1 Expedition and preserved within hydrate pressure–temperature stability conditions throughout sub-sampling and imaging processes. Measured hydrate saturation in the sub-samples, taken from units expected to have in-situ saturation of 80% or more, ranges from 3 ± 1% to 56 ± 11% as interpreted from micro-CT images. Pore-scale observations of gas hydrate in the sub-samples suggest that hydrate in silty sediments at the Gulf of Mexico is pore-invasive rather than particle displacive, and hydrate particles in these natural water-saturated samples are pore-filling with no evidence of grain-coating. Hydrate can form a connected 3D network and provide mechanical support for the sediments even without cementation. The technical breakthrough to directly visualize particle-level hydrate pore habits in natural sediments reported here sheds light on future investigations of pressure- and temperature-sensitive processes including hydrate-bearing sediments, dissolved gases, and other biochemical processes in the deep-sea environment.

## Introduction

Gas hydrate is widespread in nature^1–3^. It is especially an attractive target of commercial gas production for countries deficient in conventional hydrocarbon resources. A good understanding on the physical properties of hydrate-bearing sediments in natural reservoirs is a prerequisite to realize this goal predictably and safely. For example, acoustic wave velocities and electrical conductivities are often used to infer hydrate saturation^4–8^, which is a key parameter to determine the gas and water production potential from the reservoir^9–12^. The correlation of hydrate saturation with various physical properties highly depends on presumed pore habits of gas hydrate and inter-pore gas hydrate distribution^13–19^. However, there have been no direct observations of pore-scale hydrate occurrence in never-depressurized natural cores to support such interpretations on hydrate pore habits, mainly due to the technical complexity. This challenging task now becomes possible, as reported in this study, because of the recent development of the pressure core technique^20–29^ and high-resolution micro-CT visualization of methane hydrate^30^, including previous CT studies on pressure cores at core scale^31,32^ and high-resolution studies on THF and Xenon hydrate^33,34^ in the laboratory.

Natural gas hydrate is stable under high pressure and low temperature; therefore, it requires the preservation of the conditions consistent with gas hydrate stability throughout the coring, retrieval, and characterization. The pressure-core technique has been developed for gassy and deep sediments and used in recent gas hydrate expeditions in the U.S., China, South Korea, Japan, and India^21,22,32,35–41^. These recent efforts targeting high-saturation reservoirs have advanced the technology for pressure core cutting and transport, which is a prerequisite for this study. Petrophysical and geomechanical properties of obtained pressure cores have been extensively characterized^32,42–45^, however, with no pore-scale observations of these pressure cores.

The pore habits of hydrate in natural sediments have been inferred from various geophysical well-logging data^6,18,46–49^. Advancement in 3D non-destructive techniques including Magnetic Resonance Imaging (MRI) and X-ray CT enables direct observations of synthetic or analog hydrate crystals in sediment pores^34,50–54^. More recently, the hydrate intra-pore morphology and inter-pore distribution have been analyzed with increasing resolution^33,54–56^. Due to the limitation of the X-ray CT technique, the sample size should be sufficiently small to obtain high-resolution images, which is required to resolve high fidelity of hydrate morphology in the small pores of natural hydrate-bearing sediments. Yet, the retrieval of a small-size core while maintaining the stability of hydrate as well as the integrity of natural hydrate-bearing sediments is unprecedented.

This study involves the characterization of a 30-cm long pressure core (core H005 5FB-3: 426.51–427.63 m below seafloor), which was retrieved from the Green Canyon Block 955, Northern Gulf of Mexico^28^. The deposit is composed of interbedded sandy silt and clayey silt layers^57^. The hydrate saturation in sandy silts at this site ranges from 79 to 93% as determined by collecting the amount of gas during pressure core degassing and the pore water salinity is close to the seawater concentration^29,58^. Additional information about the pressure core such as the drilling process can be found in Ref.^28,29^. We developed a set of tools that allows sub-sampling mini-cores from the pressure core for micro-CT scans^59^, which we applied to core H005 5FB-3. Note that prior to sub-coring, the pressure core has undergone a series of events: the pressure coring was conducted in May 2017, the cores were scanned using PCATs (Pressure Core Analysis and Transfer System)^60^ and cut into 3 sections that were transferred to the University of Texas (UT)^28^ and stored at UT until September 2018. In July 2019, a 30-cm long section was sliced from a 111.5-cm section, transferred to an NETL presssure vessel, and transported to NETL. After micro-CT scanning, the sediments of depressurized cores are collected and analyzed to obtain their mineralogy and index properties and to further test any implications associated with specific non-native fluids used in the imaging procedure. This manuscript will focus on the nature and implications of pressure core degradation due to mechanical, thermal, and chemical disturbances during pressure core handling, our initial observations of pore-scale gas hydrate morphology and inter-pore hydrate distribution, and observations during gas hydrate dissociation.

## Results

### Observations during operation

**Figure 1:**
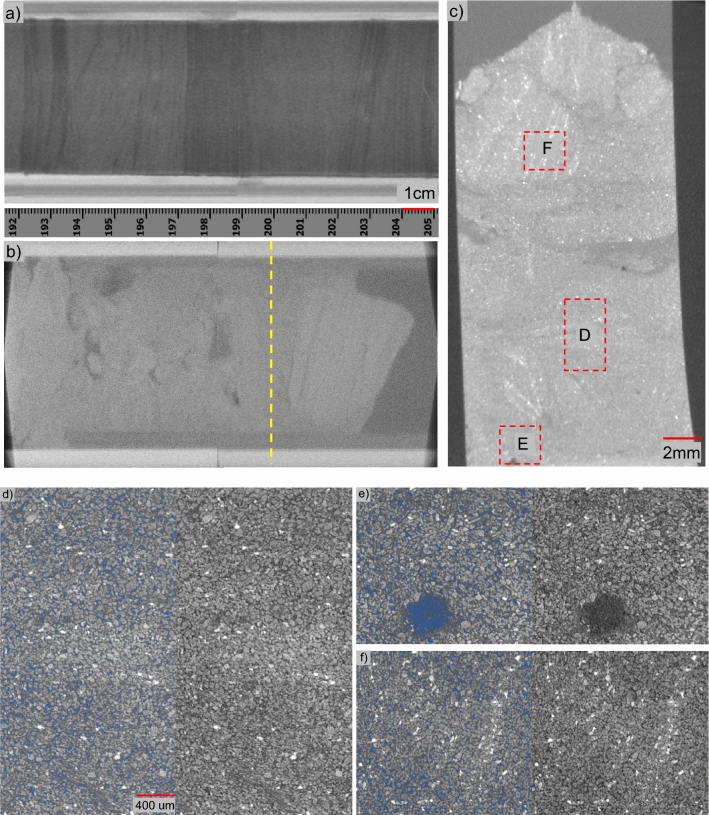
CT scan images of pressure cores at different times and scales. Figure (**a**) is a sectional view of a portion of core H005 5FB-3 scanned on board soon after the drilling of the pressure core, May 2017^29^; figure (**b**) is a sectional view of the same pressure core scanned with the industrial CT scanner (the remaining segments of the 30-cm long pressure core after three cuts), Dec 2019; figure (**c**) is an overview of the mini-core (Sample #3, Feb 03, 2020) drilled from the left-hand portion of the core shown in (**b**); figure (**d**) is high-resolution image of the portion of Sample #3 (noted as D on **c**: Jan 31-Feb 03, 2020); figure (**e**) is high-resolution image of the portion of Sample #3 (noted as E on **c**: Feb 03–05, 2020); and figure (**f**) is high-resolution image of the portion of Sample #3 (noted as F on **c**: Feb 05–07, 2020). Scales in (**a**) and (**b**) are provided by the ruler; (**d**), (**e**), and (**f**) share the same scale bar; (**d**), (**e**), and (**f**) show raw CT images on the right and the same image with hydrate false-colored in blue. Note that this false color was assigned purely according to the pixel grayscale values.

The core-scale CT scan images of the 30-cm long core at two different times, in May 2017 at UT and December 2019 at NETL respectively, are displayed in Figs. 1a and 1b. Core stratification is clearly seen in Fig. 1a and within the right-hand portion of Fig. 1b. A comparison between the two shows that the overall shape of the pressure core changed during these two scans. It is challenging to correlate the two sets of CT images due to all the events during May 2017 and December 2019 (major cause should be multiple times of handling conducted at NETL, further analyzed in section “Solid dissolution and diffusion of NaI”).

The CT scan of the pressure core in Fig. 1b was conducted while the core was placed vertically. Note that there were water pockets (darker area) in this core, suggesting some binding mechanisms among particles to prevent them from falling into the water pockets. The core in Fig. 1b was cut into two segments (separated by the yellow dashed line): the one on the right with a large chunk of the relatively well-preserved core where the mini-core Sample #2 was obtained, and the left portion where the sub-cored Sample #3 was obtained. As shown in Fig. 1b, the structural deterioration of the core in the left portion appears to be particularly severe. When this portion was laid horizontally and pushed into the sub-coring chamber, the height of the core was less than half of the plastic liner diameter. Our first attempt to get a mini-core with the drill bit failed to collect any core material. The sediments may have displayed some slurry-like behavior so that the slump of the core was very severe. We used a funnel-shaped piston to push the sediment in the following run and pushed the drill bit through to collect a mini-core.

Mini-core (Sample #3, Fig. 1c) contains, similar to the original pressure core in Fig. 1b, relatively intact blocks of hydrate-bearing sediments, as well as some heavily disturbed sediments (i.e., cores that have been separated into small agglomerations or even individual particles) during the sub-coring operation. Water pockets (darker area in Fig. 1c) are also observed in this mini-core. The pressure core changed from a vertical position during core-scale CT scanning to a horizontal position during sub-coring, which could be accompanied by core movement, and the mini-core in Fig. 1c contains a collection of fragments rather than an intact piece of pressure core, therefore we cannot exactly match Fig. 1c with b.

Figures 1d–f display high-resolution scan images of the areas within the red frames in Fig. 1c. Bright particles shown are heavy minerals (i.e., minerals with higher density or larger atomic number elements) that make them more attenuative to X-ray. Gas hydrate is false-colored blue in the CT images.

### Hydrate saturation and distribution

**Figure 2:**
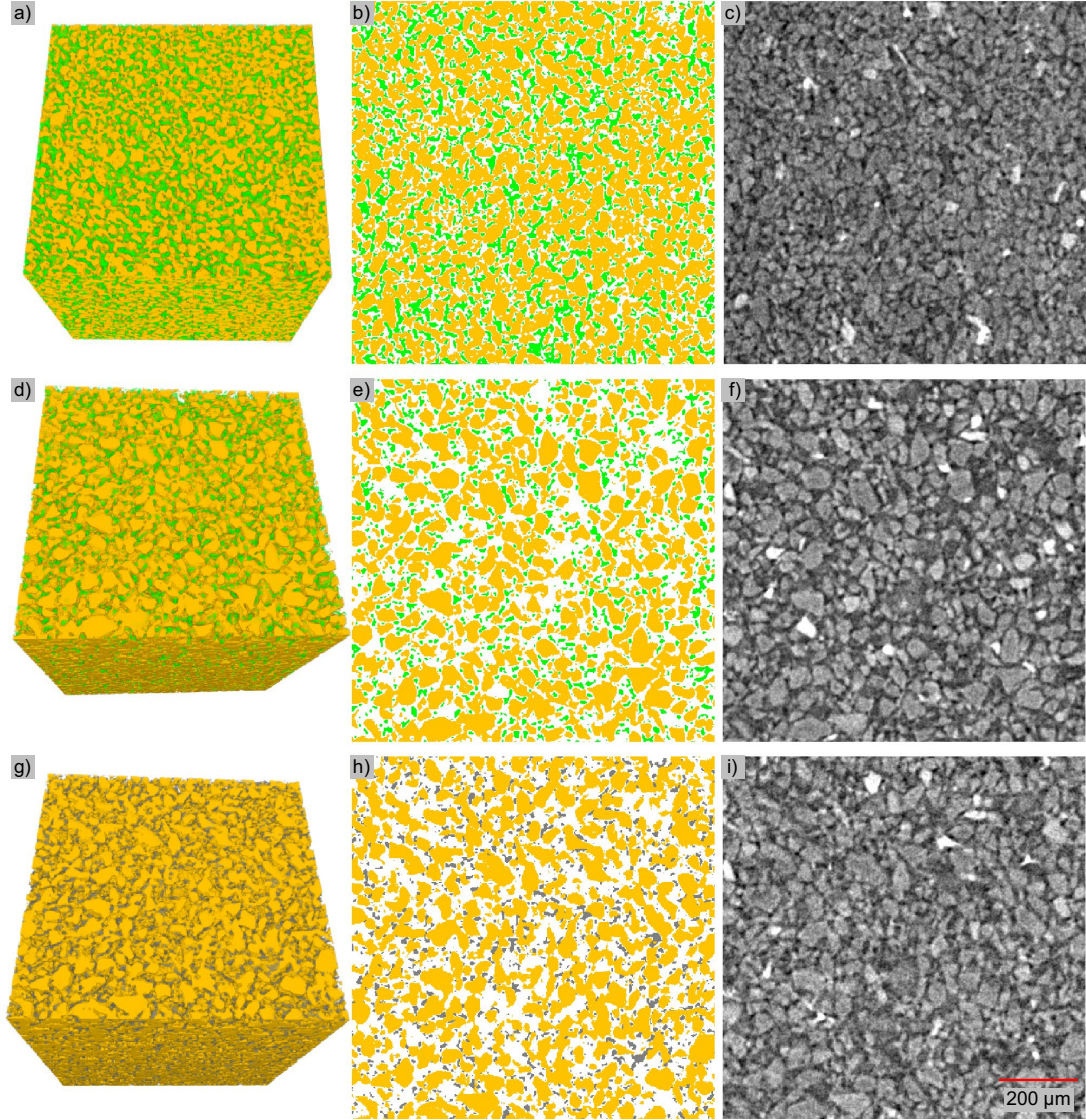
Visualization of segmented CT images. Figures (**a**–**f**) show hydrate-bearing pressure core (10 ºC, 24.1 MPa, within hydrate stability zone), (**a**–**c**) have a higher saturation than (**d**–**f**). Panels (**g**), (**h**), and (**i**) (24 ºC, 11 MPa, outside hydrate stability zone) are roughly the same area as (**d**), (**e**), and (**f**) after hydrate dissociation. Segmented results are shown in 3D (**a**, **d**, and **g**) and 2D (**b**, **e**, and **h**). Images in (**b**), (**e**), and (**h**) correspond to the top slice in (**a**), (**d**), and (**g**). Sediment particles, hydrate particles and gas bubbles are in yellow, green and gray respectively, and pore fluid is made transparent. Raw images of (**b**), (**e**), and (**h**) are shown in (**c**), (**f**), and (**i**). Sediment particles, NaI doped pore fluid, gas hydrate, and free gas are with decreasing grayscale values in (**c**), (**f**), and (**i**).

Figure 2 shows the three high-resolution scans from mini-core Sample #3: Figs. 2a–c correspond to the area F in Fig. 1c, and Figs. 2d–f and g–i correspond to the upper zone in area D in Fig. 1c. The voxel size of the high-resolution CT scan in this study is 2.3 microns, while the sizes of pores and particles are ~ 5–20 microns and 50–70 microns respectively. Note that the sample has also undergone alterations due to mechanical vibration, thermal fluctuation within hydrate stability zone, and chemical disturbances involving dissolution during sub-coring. Therefore, the hydrate visualized in this study dose not always fully capture their pore-scale morphology in-situ.

The obtained hydrate saturation in the cubic regions shown in Figs. 2a and d are approximately 45 ± 10% and 14 ± 3% respectively. If we consider the interface between sediment particles and hydrate is accurate, and voxels at the interface between hydrate and pore fluid have 25% of chance being segmented into a wrong phase, the error for the high-resolution case is ~ 10%. The error analysis is detailed in the “Methods” section. Raw CT images of these scans are also described in the “Data Availability” section to allow readers analyze and segment the hydrate phase independently.

The corresponding porosity of the two cubes is ~ 45% and ~ 49% via CT images. Note that in-situ porosity in natural reservoirs is lower due to high effective stress in the range of 3–4 MPa. Given the fact that the porosity of a reconstituted sandy silt sample from the same site reduces from 0.43 to 0.38 when in-situ effective stress is applied^61^, if considering a reduction of porosity from 45 to 40% due to in-situ effective stress, the hydrate saturation in Fig. 2a would correspond to a hydrate saturation of 56 ± 11% under in-situ stress condition.

Hydrate particles are often more angular (Fig. 2e) compared to gas bubbles in the pores (Fig. 2h), as the aspect ratio of gas bubbles is arguably less than that of hydrate. Hydrate saturation is not uniform across the sample (Figs. 1 and 2), which can be due to or exacerbated by sample degradation via thermal, mechanical, chemical, and kinetic disturbances during operation.

### Hydrate dissociation

Hydrate dissociation in all three mini-cores was initially via thermal stimulation up to 24 ºC. The corresponding pressure inside the chamber is 24.1 MPa, at which the volume of released gas is expected to be ~ 63% of the original hydrate volume, presuming mass conservation of methane during dissociation and ignoring the dissolved methane in fluids. This means the resultant gas saturation purely by thermal stimulation is lower than the initial hydrate saturation (can occur in a variety of natural sites^1^). Most of the gas bubbles were still trapped inside the sediment matrix in the samples. For Sample #3 in particular, the pressure was further reduced to 11.0 MPa (1600 psi) at 24 ºC, and the volume of released gas under this condition becomes ~ 136% of the volume of the initial hydrate. The residual gas saturation in Fig. 2g is 17 ± 4%, approximately 1.4 times the initial 14 ± 3% of hydrate saturation, which indicates that many of the gas bubbles are trapped inside the sediments after hydrate dissociation by thermal stimulation and depressurization to 11.0 MPa (Fig. 2). This agrees with earlier laboratory observations that water flows out first while gas is mostly trapped inside the pores due to capillarity under relatively high pore pressure with marginal pressure gradient. Further depressurization is required to release the gas^62^.

#### Sample deformation

**Figure 3:**
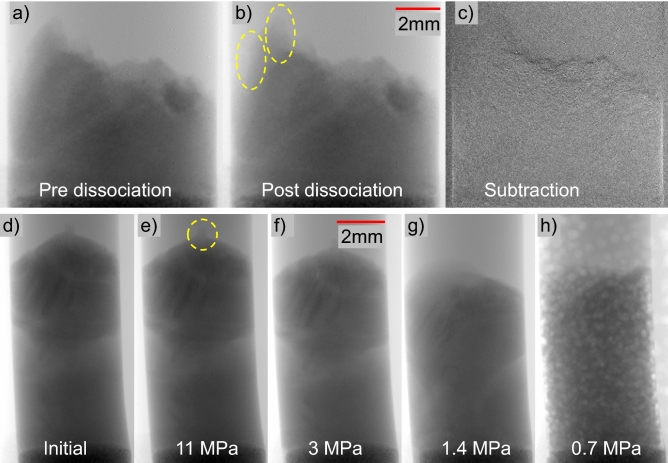
Dissociation of Samples #2 (**a**–**c**) via thermal stimulation and #3 (**d**–**h**) via thermal stimulation and depressurization. Figure (**c**) is the subtraction image between (**a**) and (**b**).

The reconstructed CT images acquired after depressurization are typically blurred, because the resultant gas bubbles are not stable in shape or position during the scanning. Therefore, we show the 2D X-ray projections instead of CT images here to demonstrate the sample deformation during the temperature increase. The sample expansion is not significant, and the sample height increased by up to 1% for Sample #2. The volume expansion of the pore fluid is not much at this pressure and temperature. At higher pressure, the total volume expansion of the pore fluid, including gas and brine/water, decreases^62,63^. Figure 3 shows the images before and after thermal dissociation (a and b) as well as the subtracted image (c). The fluid expansion during the thermal stimulation did not induce noticeable sample expansion in Sample #2. On the contrary, as dissociation and depressurization occurred in Sample #3, the sample height decreases (Figs. 3d–h). This was due to the quicker fluid flow outward, during depressurization, from the confining fluid space than that from the core space, which expanded the rubber sleeve (Fig. 3f). Therefore, the diameter of the sample increased. Large bubbles became obvious as depressurization proceeded and gas bubbles generated were able to displace the sediment particles (Fig. 3h). The results presented here strongly suggest ineffective gas production via thermal stimulation at the GOM hydrate site, and recorded sample deformations through CT scans can be a caliper for geomechanical constitutive modeling that can be expanded to predict larger scale behaviors during gas production from natural hydrate-bearing sediments.

### Sediment properties

**Figure 4:**
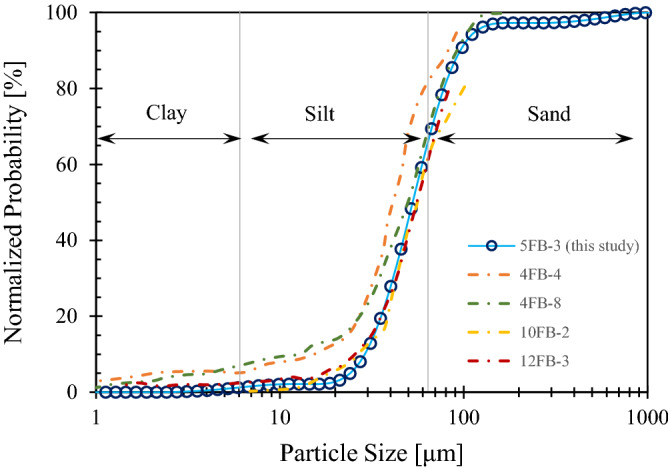
The particle size distribution of Sample #3 (5FB-3, this study) using a laser diffraction particle size analyzer. Grain size distributions of other GOM2 sediments with the same lithofacies, 4FB-4 and 4FB-8^61^, 10FB-2 and 12FB-3^57^, using the hydrometer method are also shown.

Post hydrate dissociation characterization of Sample #3 focused on particle size and surface texture, as well as their implications to hydrate and sediments behaviors. The grain size distribution of this sample was obtained using a laser diffraction particle size analyzer (Malvern Mastersizer 3000). The analysis was conducted with water dispersant and assumed non-spherical particles with silica mineralogy. The result (Fig. 4) indicates a sandy silt sediment containing 36.4% sand (> 62.5 μm), ~ 1.2% clay (< 4 μm), and approximately 62.4% silt. The tested sample has median particle size *d*_50_ = 52.8 μm, characteristic particle size *d*_10_ = 28.9 μm, the coefficient of uniformity *c*_u_ = 2.06, and the coefficient of curvature *c*_c_ = 0.99. The relative magnitude of capillarity and skeleton force in the sediments^17^ can be estimated to be *Ψ* = 10*γ*_hw_/(*d*_10_ *σ*') < 10^–3^, where the hydrate-water interfacial tension is *γ*_hw_ = 0.0032 N/m and considering in-situ effective stress of *σ*' = 4 MPa. Such a low capillarity compared to the skeleton force indicates a pore invasive hydrate pore habit, which agrees with CT observations.

The specific surface of the sediment particles passing #200 sieve (< 75 μm) is *S*_s_ = 3.0 m^2^/g, determined using the methylene blue method^64^. This value suggests the finer grains are mainly silt, the specific surface of which is typically in the order of 1 m^2^/g. Such a specific surface area of the sediments implies moderate intrinsic permeability. The hydrate accumulation in this silty reservoir is not hindered as high concentration hydrate (*S*_h_ = 79–93%) is found in sandy silt beds at Green Canyon Block 955 in the Gulf of Mexico^58^.

**Figure 5:**
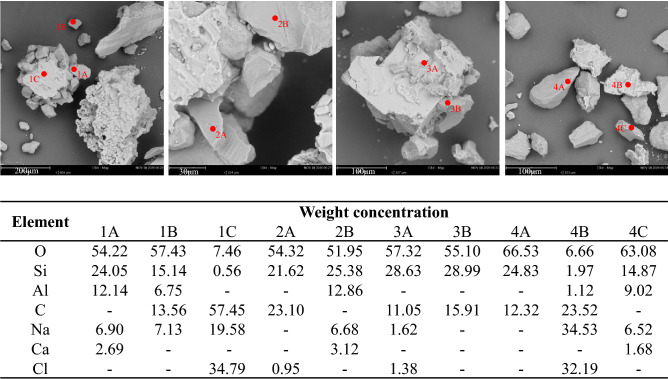
SEM–EDS analysis of the sediments at 15 kV energy. Elements at marked spots in the SEM images are also tabulated.

Energy dispersive X-ray spectroscopy (EDS) analysis shows that the major compositional elements of the sediments are silicon, aluminum, sodium, and oxygen (Fig. 5), suggesting mainly quartzitic and plagioclase minerals. The sandy silt lithofacies in other GOM2 cores from the same site also contain mainly quartz and plagioclase, with accessory minerals including potassium feldspar, dolomite, illite–smectite and calcite^57^. Representative grain surface texture is shown in scanning electron microscopic images (Fig. 5). Most grains have high relief, abrupt edges, and linear steps, indicating intensive crushing; however, no evident percussions, conchoidal fractures, or edge rounding are observed for these grains, suggesting little fluvial transport^65–67^. Therefore, the silt-sized particles in the sediments are likely the result of mechanical crushing and breakage of larger quartzitic sands mostly in-situ, with limited subsequent transport or chemical weathering. Additionally, the relatively high angularity and slenderness of the grains suggest strong anisotropy, high friction angle, and stress locking during unloading in these sediments^68–70^.

## Technical issues

### Solid dissolution and diffusion of NaI

NaI solution was used to pressurize the sub-coring and micro CT assembly. Since this solution did not contain dissolved methane or any other minerals initially, we speculate that dissolution of hydrate and minerals in pore fluids may occur. The hydrate saturations from CT observations (Fig. 2) are lower than that measured during the drilling expedition in adjacent sediments (~ 80%) with similar wave velocities^58^, which we suspect is mainly caused by hydrate dissolution. Hydrate dissolution was likely significant throughout the sample and within all sub-samples. For micro-CT mini-cores, the sediment pore volume of Sample #3 is ~ 0.5 ml, relatively small compared to ~ 11.5 ml methane-free NaI solution inside the micro-CT scanning chamber. If assuming the NaI fluids can eventually be fully saturated by methane released from dissolved hydrate, it would reduce the original hydrate saturation by ~ 20 ± 1% in the mini-core. This estimation assumes the solubility of methane with the presence of hydrate at 24.4 MPa, 6 ºC and 3.5 wt% seawater is ~ 0.07 mol/L^71^, and methane density in methane hydrate is ~ 120 g/L = 7.5 mol/L^62^. For Samples #1 and #2 with much less pore volume (~ 0.17 ml), the hydrate saturation reduction due to dissolution can be up to 65%.

Similarly, the calculation based on mass conservation shows that the NaI solution can dissolve ~ 0.92 mg or 0.46 μL of amorphous silica, considering a solubility of 80 mg/L^72^. Considering the sediment particle volume of ~ 0.5 ml and potential dissolution of other minerals, this means that ~ 0.1% by volume of the minerals has dissolved into the fluid. Such amount of mineral dissolution may not be simply ignored. Furthermore, if we consider uneven dissolution, particles closer to large quantities of operating fluid may suffer more severe loss.

**Figure 6:**
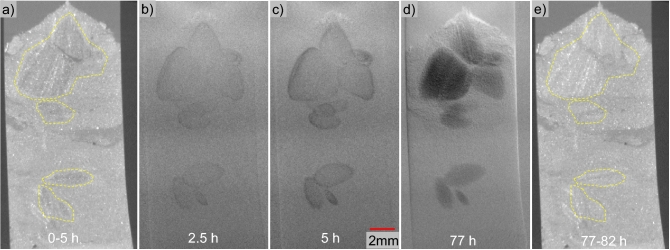
Overview CT images (**a** and **e**) and subtracted X-ray projections (**b**–**d**) showing salt diffusion in the mini-core. Note that (**a**) and (**e**) were an average of the mini-core during a period of 5 h, and (**b**–**d**) were the subtraction between the X-ray projections taken at the shown time and the X-ray projection taken at 0 h.

NaI was used as an X-ray attenuation enhancing agent, and the X-ray images, both 2D projections and 3D reconstructed CT images, can be used to track the distribution of NaI within the pore space. Figure 6 shows image slices at the same location within two sets of 3D overview CT scans taken at different times (Fig. 6a and e). The areas in the yellow lines in Fig. 6a are lighter than the same area in Fig. 6e, which was obtained 3 days after Fig. 6a. The diffusion of NaI would make these areas look brighter in 3D CT images over time, until the salt concentration becomes equilibrated across the sample. Figures 6b–d display the subtracted images of the X-ray projections obtained at the shown times from the first X-ray projection taken after transferring the mini-core. The darker color indicates higher NaI concentration caused by NaI salt diffusion into the corresponding areas, as an increase in NaI concentration would increase the X-ray attenuation. Thus, the brightness in these three CT scans demonstrates the NaI concentration progression. These areas that show traces of salt diffusion demonstrate intact sediment blocks (circled in Fig. 6a, the “intact” here means that the original fabric and pore structure are maintained), in which external make-up fluid is exchanged with original pore fluid mainly through diffusion rather than convection during sub-coring and transferring process. Figure 6c shows 5 h of diffusion and Fig. 6d shows the final state where no further diffusion takes place after 77 h. Note that the system might have reached equilibrium sooner than 77 h as X-ray projections were not taken between 5 and 77 h due to an ongoing high-resolution scan. By contrast, the rest of the mini-core mainly contains loosely packed sediment and hydrate particles with little or no preservation of their original fabric or much smaller blocks so that the pore fluid was already well-mixed with the NaI solution when the mini-core was pushed into the CT-scanning chamber. The salt diffusion started as soon as the mini-core was pushed into the micro-CT scanning assembly, and it took ~ 2 h for the first X-ray projection to be taken. Therefore, there could be smaller intact blocks of sediments that reach equilibrium within the first 2 h, then these small blocks could not be identified in Fig. 6.

### Disturbances to pressure core during characterization

Degradation of natural sediments due to mechanical disturbances are unavoidable. The degradation of pressure cores can be aggravated by also chemical, kinetic, or thermal oscillations that can lead to hydrate dissolution/dissociation, gas exsolution, mineral dissolution, dissolved salts precipitation, and so on. The packing and integrity of pressure cores may be altered during acquisition, retrieval, storage, transportation, and general manipulation. Assuming that pressure and temperature are maintained perfectly during these processes, major contributing factors on pressure core degradation include (1) contact with water not well-saturated with methane for pressure core handling and storage, which will cause hydrate dissolution into the fluid, (2) salinity change that can affect the electrochemical inter-particle forces, particularly for fine-grained sediments, (3) mechanical agitations during transportation and general manipulation, especially the vibration during pressure core cutting, and (4) temperature oscillation within hydrate phase boundary that causes small but repetitive core expansion and contraction. It is difficult to quantify the impact of each factor due to the inherent entanglement among different effects and limited information available. This section attempts to explain some of these degradation factors with our observations.

**Figure 7:**
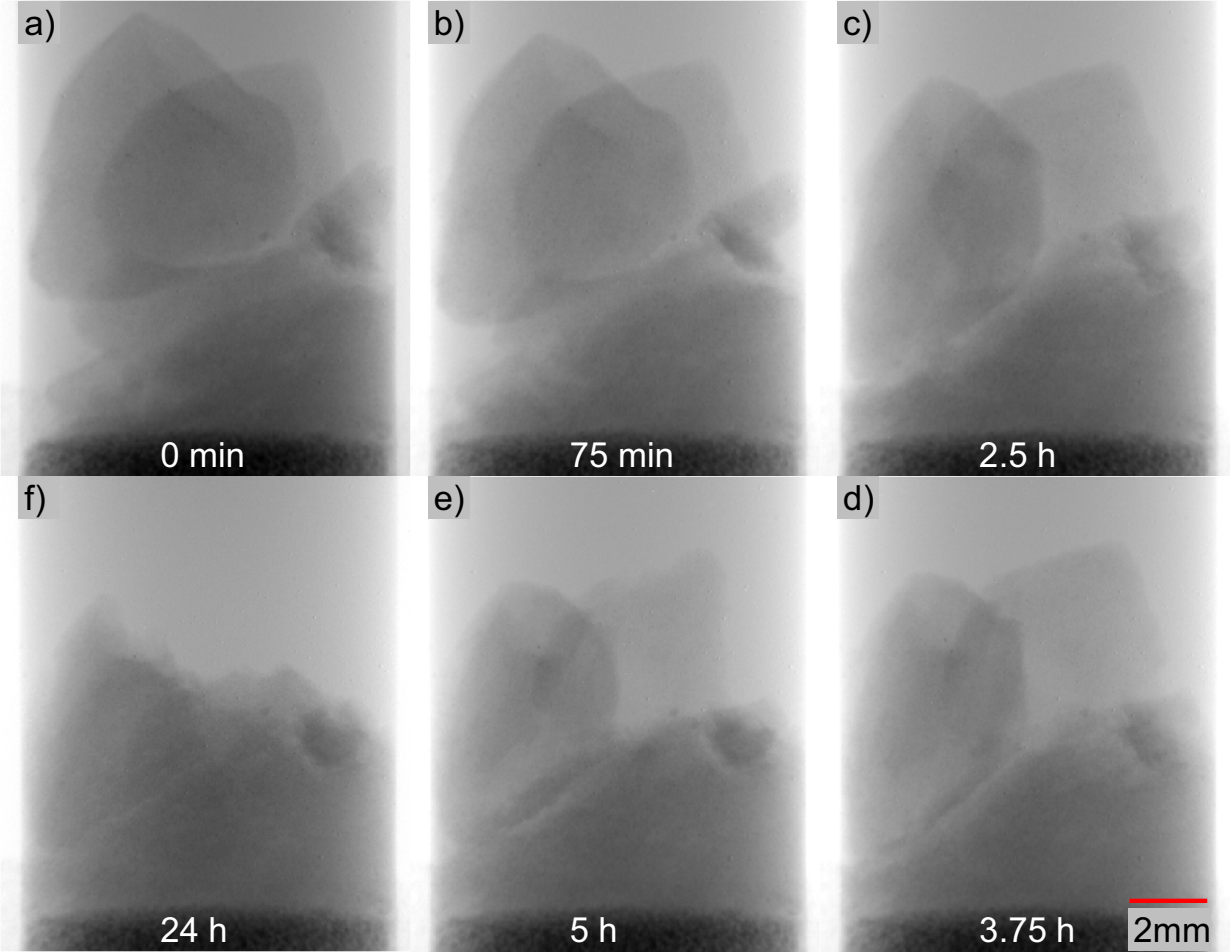
Degradation of mini-core blocks in Sample #2 in the first 24 h when exposed to the NaI solution in the core space. Since the solution was not fully saturated with dissolved methane or any minerals, hydrate and mineral dissolution into the solution may be the major cause of the collapse of this mini-core over time. See an animation in supplementary information S1 for more details.

Figure 7 shows the degradation of several intact blocks of sediments with time in the micro CT scanning chamber. The size of these blocks became smaller, and some particles detached from these blocks. This observation suggests the loss of bonding among sediment particles, possibly due to the dissolution of hydrate or minerals into the water, as there were no other disturbances such as variations of temperature or pressure nor mechanical agitation.

Samples #2 and #3 did not experience significant volume expansion during dissociation induced by thermal stimulation. Although the volume of pore fluid including both gas and brine/water expands, i.e., the total volume of released gas and water is larger than that of the initial hydrate volume, the volume expansion of the sample is not significant. In addition, the slope at the top of Sample #2 and #3 (in circled areas) is nearly vertical in Fig. 3b and e, and some blocks of the sample remained intact during this process, both of which demonstrate adhesion or stress interlocking among sediment particles after hydrate dissociation. The existence of remaining intact sediment blocks also implies that the fluid expansion was not always able to break the links among individual sediment particles nor alter the initial fabric of the sediment. Therefore, the residual strength in sediments after hydrate dissociation, which is critical to geomechanical instabilities after gas production, needs further examination considering inter-particle forces by stress locking, salt precipitation and dissolution, and electrochemical interactions.

Although the removal of gas hydrate from the sediments did cause minimal volume change in the sample, it can accompany sometimes sediment fabric change, even the breakage of cementations if any. Hydrate can be a form of cementations between sediment particles, and the fact that the sediment blocks remained after hydrate dissociation suggests there would be additional mechanisms of adhesion, although weak.

The fluid volume expansion during the subsequent depressurization was able to destroy the weak cementation. Bubbles were clearly seen growing within all three samples, pushing both sediments and the pore fluid up. The growth of bubbles was particle disruptive. By the end of depressurization, the sample became a crumbled pack of silts.

### Suggestions for future pressure core handling, testing, and visualization

#### Hydrate saturation preservation for micro-CT study

Major dissolution of hydrate in this study could be during multiple operations of the pressure core using fluids that were not saturated by methane. From the analysis of hydrate dissolution within the micro-CT scanning assembly, in the case of Sample #3, the maximum hydrate saturation loss due to hydrate dissolution can be as high as ~ 20%. This suggests that the hydrate saturation in Fig. 2d, a moderate hydrate saturation zone in Sample #3, was not higher than *S*_h_ = 34 ± 3% when it was sub-cored. Meanwhile, numerical simulations based on quasi-static diffusion (Kehua You, UT-Austin, personal communication) suggest that the dissolution mainly occurs at the periphery of the two-inch diameter pressure core and the influential zone is typically less than 10 mm if the volume of storage fluid is carefully controlled, even over an extended storage period up to two years. While the periphery of the core peels off, the center part still preserves hydrate saturation as high as > 90%^58^. Therefore, the volumes of the methane-undersaturated water, the operational time, and the number of operations prior to sub-coring should be reduced as much as possible to reduce hydrate dissolution in the pressure core. Fluids used during core transfer, cutting, and testing must be carefully selected, in terms of methane saturation, salinity, salt types, temperature, and/or dissolved minerals. It is recommended to fully characterize in-situ pore fluids and synthesize fluids with similar compositions for pressure core storage, handling, and testing. Careful control of the fluid volume and composition inside the micro-CT scanning assembly can limit hydrate dissolution during micro-CT investigations of the mini-cores.

#### Other recommendations

Mechanical agitation during sample transfer and general manipulation can disturb sediment integrity and accelerate pore fluid exchange with make-up fluids therefore should be reduced. As degradation is progressive, non-destructive measurements and imaging of pressure cores, including X-ray CT, gamma-ray density, and wave velocities, should be conducted as soon as possible upon sample acquisition. Repeat surveys can be used to gauge any degradation. Techniques to quantify the influencing factors of pressure core degradation due to physical and chemical disturbances are needed.

## Discussion

Major issues such as hydrate pore habit are discussed here, and additional analyses on hydrate-sediment interaction during mechanical agitation and the impacts of salinity on the sedimentation process are contained in supplementary information Text S1 and S2.

### Non-uniform hydrate distribution

Hydrate dissolution reduces the amount of hydrate mass and will make original hydrate particles within the pressure core smaller. Hydrate dissolution is likely not uniformly occurring throughout the same sample, as hydrate near the peripheral boundaries of ‘intact’ blocks dissolves into the surrounding water earlier and faster, while hydrate at the center portion of ‘intact’ blocks may be preserved longer. In addition, heterogeneous pore structure may contribute to the non-uniform hydrate dissolution.

### Pore-invasive hydrate

**Figure 8:**
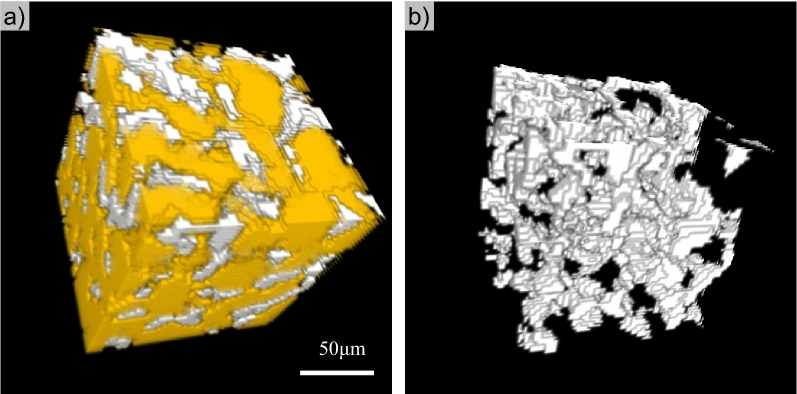
Hydrate crystals in neighboring pores are connected (1/64 of Fig. 2a). (**a**) Hydrate (white) in sediment grains (yellow). (**b**) Segregated hydrate phase in (**a**).

Natural gas hydrate in these mini-cores appears pore-invasive rather than particle-displacive. Pore-invasive refers to hydrate formation invading into the original pore structure without significantly altering the sediment fabric, while particle displacive refers to the pattern that hydrate displaces sediment particles as it forms^73^. Particle displacive hydrate is typically manifested as veins, lenses or chunks, which were not observed or suggested at such a fine scale in the mini-cores. In regions with sufficient hydrate distribution and grain size, hydrate crystals in a pore are connected to hydrate in neighboring pores through the pore throat, providing additional mechanical strength to the sediments without direct cementation. Figure 8 shows a Section (1/64 of Fig. 2a) from Sample #3 where hydrate appears to form this connected skeletal framework (See its 3D structure in supplementary information, Animation S2). As a side note, the connected hydrate mass has less interface area with its surrounding environment than dispersed individual hydrate particles, and therefore is more stable. Hydrate formation shifts from pore-invasive to particle displacive as sediment particle size and effective stress decreases^17,74,75^, and the shift occurs at a particle size of ~ 20 microns under a low effective stress condition^76^. The particle size in these natural pressure cores is almost exclusively larger than 20 microns (Fig. 2), and the much higher in-situ effective stress level would facilitate the formation of pore-invasive hydrate.

### Grain-coating or pore-filling?

Grain-coating is not observed in the micro-CT images. Although we cannot exactly quantify the contact angle of hydrate on the surface of sediment particles due to the limited resolution, the sediment surfaces generally favor water rather than hydrate in these samples (Figs. 2b, c, e and f, Fig. 8, also supplementary information Animation S2, and raw CT data). Such affinity of sediments for water would inhibit the formation of grain-coating hydrate rather than pore-filling hydrate, as observed in previous laboratory-synthesized hydrate-bearing specimens^54,77^. Note that the pore-filling here does not mean hydrate particles floating in the center of pores without touching pore walls. All hydrate particles have to be in physical contact with sediment particles, even simply to counteract the buoyancy and to prevent the upward floating of hydrate particles^78^. Note, by touching, we mean the requirement to establish a mechanical balance of hydrate particles.

## Conclusions

We conducted for the first time sub-coring of natural pressure cores and micro-CT scanning of the obtained mini-cores in this study. There are various factors such as hydrate dissolution and mechanical agitation that deteriorated the quality of the samples, which were kept within the hydrate stability zone throughout the testings. Hydrate saturation from micro-CT observations ranges from 13 ± 3 to 56 ± 11% in this study, which is considerably lower than the wave-based estimation of up to 93% hydrate saturation in adjacent sediments, mainly due to hydrate dissolution largely during the operations associated with the micro-CT sample preparation and imaging.

Observed hydrate phase within sediments pores is pore-filling and no evidence of cementing hydrate is found in the sediments from the GC955 Gulf of Mexico. Hydrate formed in these silty sediments is most likely pore-invasive rather than particle displacive at the fine spatial scales.

In the scanned sample with the highest hydrate saturation, hydrate is observed to connect adjacent pores via continuous hydrate structure bridging pore throats. This provides mechanical support to the sediment structure without direct cementation. These results set the benchmark for the past and future laboratory synthesized hydrate-bearing samples in terms of hydrate intra-pore and inter-pore morphology.

The hosting sediments are angular with surface textures of sharp abrupt and steps, indicating particle breakage in-situ and suggesting high friction angle and strong stress locking during unloading.

Fluid expansion during the thermal stimulation did not induce noticeable sample expansion with negligible effective stress in this study. Large bubbles became obvious as depressurization proceeded and expanded gas bubbles were able to displace the sediment particles under low effective stress conditions. Gas production via thermal stimulation is ineffective at this site due to a significant amount of trapped gas in sediments. Post hydrate dissociation sediments still preserve certain fabric and structural integrity, demanding a holistic examination of particle-level forces that is critical to understand the reservoir geomechanical instability after gas production.

This study detailly documents all processes of pressure core manipulation and micro-CT scanning, particularly the drilling of mini-cores and pore fluid doping with NaI salt to enhance the phase-contrast during CT scanning. This gives the first-hand experience of such work that can inspire future work on pressure core visualization, scientific explorations of gas production and geomechanical analysis using natural samples, and other pressure- and temperature-sensitive biogeochemical processes in the deep-sea environment.

## Methods

The general operations sequence and micro-CT scan procedures are described below, including sub-coring, X-ray CT scanning, hydrate dissociation, and sediment characterization. The core was transferred from the transportation chamber to a manipulator after receiving and used for testing cutting and subcoring tool three times in the following three months. The remaining portion of ~ 13.5 cm in length was transferred to the CT scanning chamber in December 2019. All these events occurred under a pressure of no less than 20.7 MPa (3000 psi) and a temperature of 6 °C with fluctuations of no more than 3 ºC during the transportation and less than 0.5 ºC during storage. In addition, all of these events were conducted with the core bathed in fluids undersaturated with respect to methane.

### Sub-coring of pressure core

#### Tools

The Pressure core Characterization and X-ray CT visualization Tools (PCXT) were developed for pressure core testing and imaging. This set of equipment comprises two groups of tools: (1) core processing tools such as a manipulator, a cutter, four 30-cm long transport chambers, and a sub-coring chamber, and (2) core analysis tools including a core-scale X-ray CT scanning chamber, a micro-CT scanning assembly, an effective stress chamber, and a triaxial testing chamber^59^.

#### Pressure core analysis operation procedure

**Figure 9:**
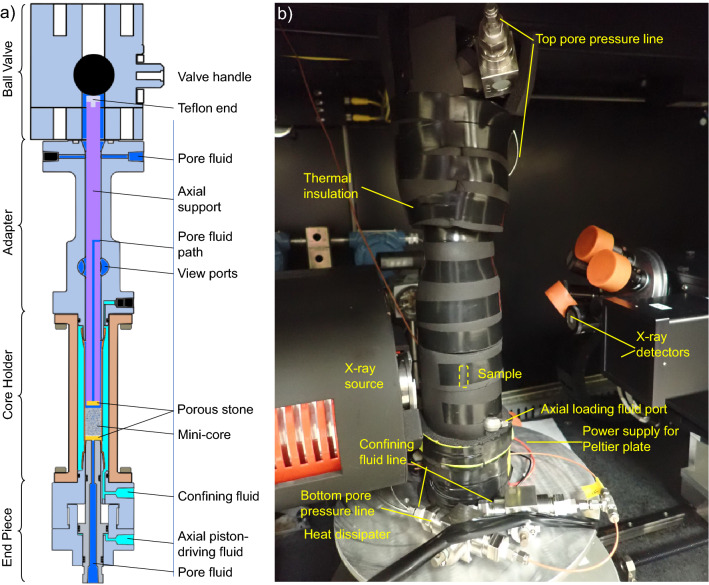
Micro-CT scanning assembly mounted on the rotary stage within the CT scanner (modified from Ref.^59^). (**a**) a vertical sectional view of the assembly; (**b**) the assembly containing a mini-core prior to micro-CT scan.

The PCXT was operated inside an environmental chamber with a constant temperature of 6 ºC, and the pressure was maintained hydraulically at 24.1 MPa (3500 psi) on the pressure core. The initial 30-cm long 50.8-mm diameter pressure core was first cut into short segments (3.8–10 cm) and transferred into the sub-coring chamber, which was then disconnected from the manipulator and connected to the sub-coring tool and the micro-CT scanning assembly (Fig. 9). Before being connected to the sub-coring tool, the micro-CT scanning assembly was pressurized to 24.1 MPa (3500 psi) via an ISCO syringe pump through two ports that connect the core space and the confining fluid inside the micro-CT scanning chamber. This guarantees that there is no pressure difference between the pore fluid and confining fluid, therefore there is no effective stress applied on the sediment skeleton during scanning. Then the sub-coring tool drilled out a mini-core of 9 mm diameter, which was finally pushed further into the micro-CT scanning assembly. The assembly was then mounted onto the sample stage of the micro-CT scanner (Fig. 9b).

We attempted to obtain a mini-core three times guided by the CT image obtained right after the offshore retrieval of the pressure cores from the reservoir (Fig. 1a). Sample #1 was obtained in August 2019 with ~ 6 mm in length, which is much shorter than the targeted 20 mm length. The images from Sample #1 are not shown due to highly altered sample integrity. After the initial attempt, a core-scale CT scan was conducted on the remaining 5FB-3 core before further sub-coring (Fig. 1b) to ascertain the current state of the pressure core. Two other mini-cores, Sample #2 and #3, were successfully obtained in December 2019 and January 2020 in subsequent attempts.

#### Water used during the operations

The space inside the micro-CT scanning assembly is separated by a rubber sleeve into two parts: the core space that hosts the sample and the confining space for applying confining pressure. While deionized water was used for large core (50.8 mm in diameter) operations, 7 wt% NaI solution was used for the mini-core inside the rubber sleeve in order to enhance the contrast between pore water and gas hydrate in micro-CT scans. Note that the fluid inside the micro-CT scanning assembly was static, and there was no flush through of NaI solution. The intent was to increase the NaI content in the sediment pores slowly via diffusion. The concentration of 7% is higher than the ~ 5% as the optimum content to enhance the image contrast, considering the fluid mixing with freshwater during the mini-core sub-coring. NaI was used for two reasons: (1) I^-1^ increases the attenuation coefficient of the pore fluid to enhance the image contrast between water and methane hydrate in the CT images, and (2) Na^+^ rather than K^+^ reduces potential chemical disturbance to the clays in the sediment, as Na^+^ prevails in natural marine environments while K^+^ is able to cause clay contraction^79^. The molarity of 7 wt% NaI solution is ~ 0.046 mol/L, lower than that of seawater ~ 0.06 mol/L. We consider the molarity of the pore fluid in the mini-core is between that of deionized water and seawater since deionized water has been used in sub-sampling operations. The salt content in the pore fluid would shift the phase boundary to a lower temperature; since there is no published data on NaI, we use seawater with the same molarity for approximation, the temperature depression of 0.046 mol/L seawater is ~ 1.1 ºC. Deionized water, rather than 7% NaI solution, was used as the confining fluid in the micro-CT scanning assembly, so that the confining fluid attenuates less X-ray energy during the micro-CT scannings.

### Micro-CT scan of mini-pressure cores

#### Pressure and temperature control

The temperature of the mini-core was controlled with a Peltier plate that extracts heat from the micro-CT scanning assembly and dissipates the heat via a heat sink. An adapting base, customized to mount the micro-CT scan assembly to the rotary stage of the micro CT scanner, was prechilled to ~ 4.5 ºC before the mini-core transfer, so that heat transferring from the CT stage to the mini-core can be reduced during the assembly mounting. The final equilibrium temperature of the mini-core was set at ~ 10 ºC. Upon the completion of the assembly mounting and thermal insulation application, 24.1 MPa (3500 psi) of backpressure and confining pressure were applied, resulting in zero effective stress to avoid sample breakage.

The micro-CT scanning assembly is able to apply confining pressure and axial load to the mini-core as illustrated in Fig. 9 (see more details in^59,80^). The mechanical load was not applied in this study mainly due to the low integrity of the mini-core (further discussed in  “Solid dissolution and diffusion of NaI” section). We therefore only conducted micro-CT scanning of the mini-core and monitored hydrate dissociation.

#### Types of X-ray scans and images

The term CT refers to computed tomography, which indicates the results are computed from a series of X-ray projections taken at different angles of a target object. Therefore, CT images are naturally in 3D, even though 2D slices cut from different angles within the 3D data set are often shown. By contrast, 2D X-ray projections are directly taken by the detector without any further computing, and the whole 3D object is projected into a 2D image. The acquisition of one set of 3D CT images requires inverse problem solving based on a large number of X-ray projections, therefore is typically time-consuming; while 2D X-ray projections can be taken at a higher frequency to track dynamic processes.

There are three types of 3D X-ray CT scans in this study: the core-scale CT scan conducted by an industrial CT scanner, the overview CT scan of the mini-core, and high-resolution scans of regions of interest within the mini-core (see Table 1). The latter two were conducted using a micro-CT scanner (Xradia XCT-400) with two different X-ray detectors. Subtraction between X-ray projections taken at different times is sometimes used to demonstrate progressing physical processes within the chosen area of view in the mini-core.

**Table 1:** X-ray image types.

Term used	Time/image	Resolution	Scanner	Detector
3D Core scale CT scan	2 h	60 μm	Industrial CT	Flat panel
2D X-ray projection	10 s	10 μm	Micro CT	0.4X
3D Overview CT scan	5 h	10 μm	Micro CT	0.4X
3D High-resolution CT scan	> 72 h	2.3 μm	Micro CT	4X

#### CT scan procedure

An overview CT scan was conducted first with a resolution of 20 microns to choose regions of interest for higher resolution micro-CT scans. The overview and the high-resolution scans take 5 h. Given the reported pore size distributions for GC955 gas-hydrate-bearing sandy silts of 2–10 microns (~ 5 microns mean; based on mercury intrusion tests—see^61^), high-resolution 3D scans were then designed to achieve the maximum feasible resolution of 2.3 microns. The longer CT scan time of > 72 h corresponds to the longer exposure time for each projection and/or a larger number of projections, which could both decrease the signal-to-noise ratio and increase the image quality. Multiple high-resolution scans were conducted for each mini-core, depending on the size of the mini-cores. Details on the CT scan configuration and the X-ray transparent core holder have been previously reported in detail^30,80^.

Note that the pore size as interpreted by mercury intrusion tests is actually pore throat size, and that those measurements represent average values over large areas of sediment. Therefore, the actual pore size, particularly if limited to small local areas of greater grain size, can be larger than 5 microns. Nonetheless, the visualization of features in the range of 5 microns given a resolution of 2.3 microns is technically challenging. Higher resolutions than we have achieved here will be required to better visualize the majority of pores within these sediments.

#### CT image segmentation

We segmented the CT image with a deep-learning based program *ilastik*^81^, which has been proven to be efficient and accurate for phase segmentation^82^. The histogram of the CT image does not show distinct peaks for different phases, therefore, simple thresholding according to voxel intensity is not viable and we use manual input to train the algorithm first before segmentation. Like how a typical machine-learning approach works, we train *ilastik* with manual input, let it run and check the result, add more manual input and check again, until we are satisfied with the result. Note that we cannot directly modify the result in this approach, and the final result is solely dependent on our manual input. Manual input considers several aspects: (1) the intensity generally decreases from sediment particles to pore fluid to methane hydrate, (2) the boundary between sediment particles and pore constituents is enhanced by X-ray diffraction, and (3) check adjacent slices back and forth before each manual input in the 3D image. Supplementary information Figure S5 shows one example of the manual input during 3D image segmentation.

#### Error analysis for hydrate saturation via segmentation

**Figure 10:**
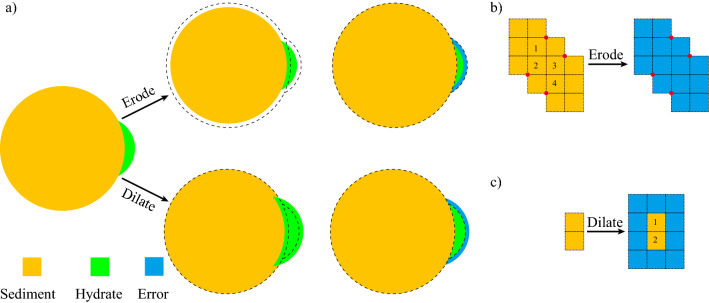
Error analysis for hydrate saturation. (**a**) approaches depiction, (**b**) and (**c**) overestimated surface areas that can fall into the category of error during “Erode” and “Dilate” operations.

The error analysis is based on the segmented image. Since the contrast between sediment particles and gas hydrate is much better than that between gas hydrate and pore fluid, we consider the error during segmentation mainly occurs at gas hydrate-pore fluid boundary. As shown in Fig. 10, we used two approaches to estimate the error. In the first approach, the function of “erode” in ImageJ is used on the combination of sediment particles and hydrate particles, Boolean operations are used to remove the change on sediment particles, and the surface area at gas hydrate-pore fluid boundary with a thickness of one voxel can obtained. In the second approach, the function of “dilate” in ImageJ is used on hydrate particles, and similarly, Boolean operations enable the calculation of the surface area. Note that the surface area obtained in the first approach is within segmented hydrate regime, while that in the second approach is within segmented pore fluid regime. If the hydrate particles are depicted with high resolution, these two approaches are close to each other and accurate enough. However, when the object, which is hydrate particle in this context, is not represented with enough resolution, these two functions in voxelized images can result in overestimation in errors. A hydrate particle with 14 voxels can disappear after erode (Fig. 10b), because all voxels are in contact with the outside region, even if the contact is via one single node (shown in red); similarly, a hydrate with two voxels can be coated by 10 voxels during dilate (Fig. 10c). Such approaches are aggressive therefore overestimate the interfacial area between gas hydrate and pore fluid. In addition, it is not likely to mistakenly classify all the surface area into a wrong phase. Overall, we consider the 25% of the voxels can be wrong, and the resulted error is reported in the text.

### Controlled dissociation of natural methane hydrate

Hydrate dissociation in all three samples was triggered via temperature increase to the ambient temperature of 24 ºC by turning off the Peltier plate while maintaining the pressure of 24.1 MPa; we then decreased the pressure gradually to atmospheric pressure. As a reference, the phase boundary of methane hydrate in fresh water at 24.1 MPa is 20 ºC, and can be shifted to ~ 19 ºC due to the salt content considering the ~ 1.1 ºC of temperature depressions. Complete dissociation of hydrate occurred over 40 min. For Samples #1 and #2, the micro-CT scanning assembly was left for equilibrium at 24.1 MPa and 24 ºC for additional two hours before we conducted a 3D overview CT scan and a high-resolution 3D CT scan. For Sample #3, the overview and high-resolution 3D CT scans were conducted at a later stage when the pressure was lowered to 11 MPa at 24 ºC. 3D high-resolution CT scans at two resolutions were conducted again when the pressure was down to 0.7 MPa for all three samples. The thermal dissociation and depressurization processes were monitored via real-time 2D X-ray projections for all samples.

### Post-dissociation sediment characterization

The depressurized Sample #3 was further characterized for index and physical properties. Tests conducted on the sediments recovered from the dissociated mini-core include (1) particle size distribution using a laser diffraction particle size analyzer (Malvern Mastersizer 3000); (2) the sediment specific surface using the methylene blue method; (3) energy dispersive X-ray spectroscopy (EDS) analysis to assess the mineralogy of the sediments; and (4) scanning Electron Microscope (SEM) images to facilitate analyses based on the particle shape and surface texture.

## Supplementary Information

Supplementary Information 1.

Supplementary Information 2.

Supplementary Information 3.

Supplementary Information 4.

## Data Availability

Raw CT data as well as the segmented CT data is shared via EDX run by National Energy Technology Laboratory (https://edx.netl.doe.gov/dataset/gas-hydrate-pressure-core).
