# Supplementary material for: Pore-scale observations of natural hydrate-bearing sediments via pressure core sub-coring and micro-CT scanning
Source: Sci Rep. 2022 Mar 2;12:3471. doi: 10.1038/s41598-022-07184-6 (PMC8891283; doi:10.1038/s41598-022-07184-6)
Supplement: Supplementary file 1 — Supplementary Information 1. [file 41598_2022_7184_MOESM1_ESM.docx]

**Introduction**

This supporting information contains some animation files that either show the evolution of a mini-core with time (Figure S1) or assist the visualization of 3D hydrate morphology (Figure S2) and two discussion points (Text S1 and S2, Figures S4 and S4) related to hydrate-sediment interaction during mechanical agitation and the impacts of salinity on the sedimentation process of the micro-CT core recovered from Green Canyon Block 955 during UT-GOM2-1.

Figure S1. Pressure core degradation with time shown in X-ray projections. This animation complements Figure 7 in the main text.

Figure S2. 3D structure of a small piece of segmented pressure core. a) 3D structure of sediment and hydrate with pore fluid made transparent, and b) 3D structure of hydrate only. These animations complement Figure 8 in the main text.

Text S1. Geometric entrapment of hydrate particles

Hydrate particles are occasionally found in the mini-core where sediments are heavily degraded into a crumbled stack (Figure 2d). If hydrate particles are attached to the sediment particles, the presence of hydrate particles in degraded sediments can be easily explained as falling sediment particles during degradation can bring the attached hydrate particles together. By contrast, hydrate particles would be able to float up due to buoyancy under certain conditions and if there is no bonding. A discrete element method (DEM) simulation is performed to assess the possibility of geometric entrapping of floating hydrate particles in degraded sediments in an idealized low hydrate saturation case. Sediment particles are simulated by perfect spheres, and particle shape effect or stress interlocking associated with angular particles is not the focus. Two kinds of particles are used in the simulation: heavy particles with a density of 2.65 and light particles with a density of 0.93, representing sediment and gas hydrate particles respectively. Simple cubic packing of sediment particles with a diameter of 100 microns was used in the initial state to form a cubic sample, and each pore was filled with one hydrate particle with a diameter of 73 microns (maximum diameter of a ball to fit in the pore). This sample was placed inside a tube (Figure S3). This sample was allowed to collapse into a pack of particles after removing the confinement to represent mechanical disturbance. Then the tube is tilted by 90 degrees to allow particles to slide along the tube wall, simulating the operation where the micro-CT scanning assembly was initially horizontal but later verticle during micro-CT scans.

Results show that even if there is no bonding between sediment and hydrate particles, there are still hydrate particles trapped inside the sediments: 24.8% after the collapse and 0.8% after tube tilting within a cubic sample with a size of 1.2 mm^3^. These two ratios increase to 38.1% and 2.0% respectively as the size of the cubic sample increases to 1.8 mm^3^. At higher hydrate saturation levels such as in Figure 8, hydrate particles can form a network that traps sediment particles, and form small blocks of composite structure without cementation between hydrate and sediment particles. In both cases, we cannot easily exclude the possibility that there may not be any bonding between hydrate and sediment particles even if they appear to be contacting each other.

Figure S3. Discrete element method simulation of hydrate-bearing sediments degradation and slippage. (a) Initial condition in the simulation, sediment and hydrate particles are in red and green confined within a cube; (b) sediment particles in the cubic specimen collapse after removing the boundary confinement while some of the hydrate particles were able to escape from the matrix and float up to the top of the container; (c) more hydrate particles escape from the sediment matrix when the tube is tilted, and (d) the final state of the simulation when the tube is placed vertically.

Text S2. Sedimentation porosity

The fabric and behavior of clayey and silty sediments are largely influenced by pore fluid pH and ionic concentration, which govern the double layer thickness, the surface charges, and the fabric of fine particles. Sedimentation tests are easy to perform and can be insightful to understand interparticle forces [[Palomino and Santamarina, 2005](#_ENREF_45); [Patton, 1964](#_ENREF_47); [Pierre et al., 1995](#_ENREF_50)]. The final settlement volume reveals the state of dispersion and particle to particle interactions. In this study, approximately 3 grams of oven-dried fine-grained particles were mixed with fluid in a glass graduated cylinder to form the sedimentation suspension. The fluid was de-aired water with various salt concentrations (sodium chloride, c = 0, 0.17, 0.34, 0.6, and 0.85 mol/L). To contrast the impact of surface charges, sediment surface covered by methylene blue was also used in the sedimentation tests. Methylene blue in the water is a cationic dye C16H18N3S+ that is absorbed to negatively charged particle surfaces to determine surface area and neutralize the surface charge [[Hang and Brindley, 1970](#_ENREF_18)]. The graduate with suspensions was repeatedly inverted to enhance the mixing until a uniform appearance was observed. Then the graduate was placed on a level surface to start the sedimentation process. The results in Figure S4 show that the final sedimentation porosity increases with increased salt concentration for both sediments, and the sediments with electrically neutralized surfaces (i.e., covered by methylene blue) render a much lower porosity. Increased final sedimentation height and slowed sedimentation velocity with increased ionic concentration are also observed in kaolinite at pH = 3 and 7 [[Palomino and Santamarina, 2005](#_ENREF_45)]. Note that the salt concentration in seawater is around c = 0.6 mol/L, and hydrate dissociation generates freshwater that reduces the salt concentration in pore water (i.e., pore water freshening). Therefore, particles in these sediments are prone to contract in volume and agglomerate into larger flocs when hydrate dissociates during gas production.

Figure S4. Sedimentation porosity in water with different salt concentrations.

**Figure S5.** One example of manual input to train the algorithm during image segmentation. a) Raw image, b) manual input for Sand, NaI solution and hydrate as shown in the circles, c) probability output, and d) segmented image.
