# Supplementary figures and images for: Pore-scale observations of natural hydrate-bearing sediments via pressure core sub-coring and micro-CT scanning
Source: Sci Rep. 2022 Mar 2;12:3471. doi: 10.1038/s41598-022-07184-6 (PMC8891283; doi:10.1038/s41598-022-07184-6)

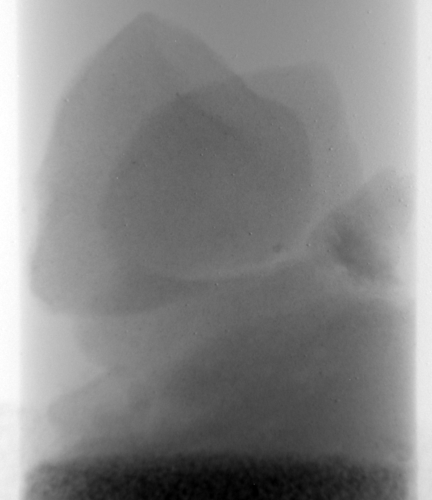

Supplement: Supplementary file 2 — Supplementary Information 2. [file 41598_2022_7184_MOESM2_ESM.gif]

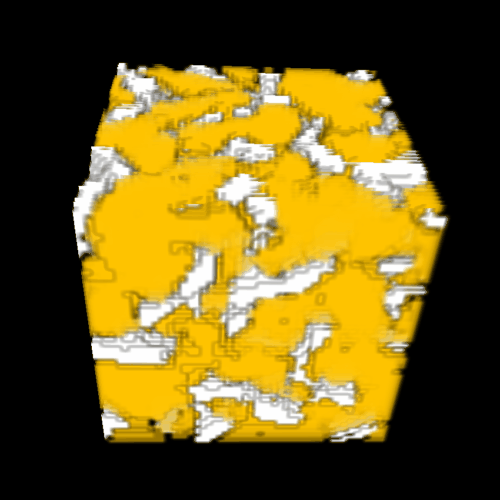

Supplement: Supplementary file 3 — Supplementary Information 3. [file 41598_2022_7184_MOESM3_ESM.gif]

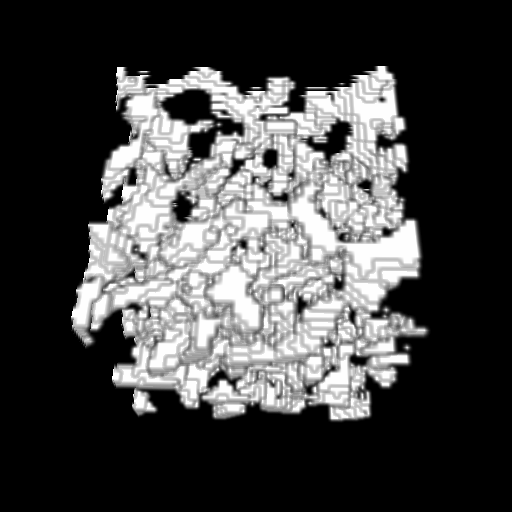

Supplement: Supplementary file 4 — Supplementary Information 4. [file 41598_2022_7184_MOESM4_ESM.gif]
